# Recombinant GII.P16-GII.2 Norovirus, Taiwan, 2016

**DOI:** 10.3201/eid2307.170212

**Published:** 2017-07

**Authors:** Luke Tzu-Chi Liu, Ting-Yu Kuo, Ching-Yi Wu, Wan-Ting Liao, Aron J. Hall, Fang-Tzy Wu

**Affiliations:** Centers for Disease Control, Taipei, Taiwan (L.T.-C. Liu, T.-Y. Kuo, C.-Y Wu, W.-T. Liao, F.-T. Wu);; Centers for Disease Control and Prevention, Atlanta, Georgia, USA (A.J. Hall)

**Keywords:** Norovirus, GII.2, acute gastroenteritis, outbreak, viruses, Taiwan, enteric infections

## Abstract

In Taiwan, acute gastroenteritis outbreaks caused by a new norovirus genotype GII.2 increased sharply toward the end of 2016. Unlike previous outbreaks, which often involved restaurants, GII.2 outbreaks mainly occurred in schools. Phylogenetic analysis indicates that these noroviruses are recombinant GII.P16-GII.2 strains.

Norovirus, the leading global cause of epidemic gastroenteritis, is responsible for >90% of all viral gastroenteritis and ≈50% of gastroenteritis outbreaks worldwide (*1*). In Taiwan, variants of the genotype GII.4 had been the most prevalent genotype in norovirus-associated acute gastroenteritis outbreaks until mid-2014 (*2*). Since winter 2014–15, a previously uncommon genotype, GII.17, became the predominant genotype in Taiwan. This GII.17 strain also caused many outbreaks in Japan and was reported sporadically in other parts of the world (*3*,*4*). Since September 2016, we have identified a new GII.P16-GII.2 strain that has replaced GII.17 as the predominant strain in norovirus-associated outbreaks in Taiwan, concurrent with a sharp increase in GII.P16-GII.2 norovirus outbreaks and sporadic cases during the 2016–17 season in Germany (*5*).

## The Study

**Figure 1:**
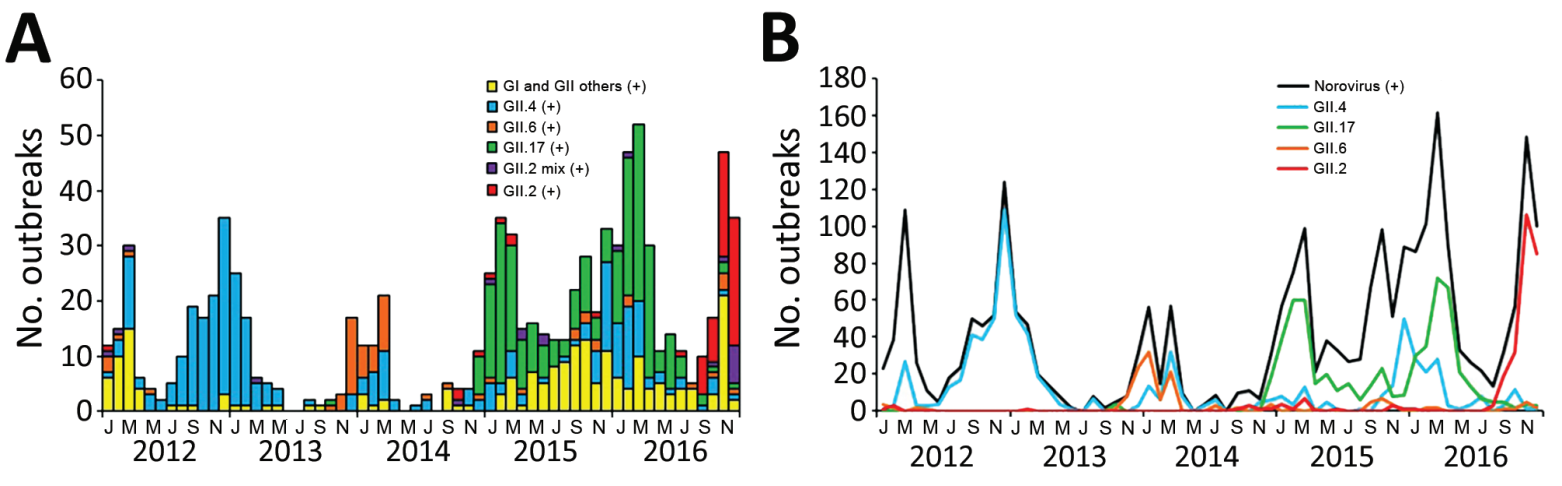
Reported monthly norovirus outbreaks, Taiwan, 2012–2016. A) Outbreaks caused by seasonal predominant strains (GII.2, GII.2 mixed with other genotypes in the same outbreak, GII.17, GII.6, GII.4, and other GI and GII genotypes). B) Monthly trends of all norovirus and predominant norovirus genotypes. +, positive.

During January 2015–December 2016, a total of 876 acute gastroenteritis outbreaks were reported to the Centers for Disease Control in Taiwan. A total of 576 (65.8%) outbreaks were identified as norovirus by reverse transcription PCR with primer pairs G1SKF/G1SKR and G2SKF/G2SKR for GI and GII genogroup detection, respectively; all positive samples underwent sequence-based genotyping by using the Norovirus Genotyping Tool (Table 1) (*2*,*6*,*7*). Before August 2016, GII.2 strains had been detected only sporadically (Figure 1). By September 2016, GII.2 accounted for 60% of norovirus-associated acute gastroenteritis outbreaks that month and, in December 2016, for 86%. Globally, GII.2 is an uncommon genotype that accounted for ≈1.2% of sporadic norovirus infections in children during 2004–2012; by contrast, GII.4 was responsible for 67.2% of norovirus infections during the same period (*8*). In 2016, the settings of outbreaks involving GII.2 differed from those of non-GII.2; most (72.0%) GII.2 outbreaks occurred in schools, whereas most non-GII.2 outbreaks occurred in restaurants (41.4%) and fewer occurred in schools (31.0%). Within schools, half of GII.2 outbreaks occurred in kindergartens, 33.3% in elementary schools, and 14.9% in junior/senior high schools and colleges/universities (Table 2).

**Table 1:** Norovirus detection in acute gastroenteritis outbreaks, Taiwan, 2012–2016

Detection	Outbreaks, no. (%)				
2012	2013	2014	2015	2016
Total norovirus	175 (57.8)	80 (32.1)	76 (28.8)	262 (60.9)	314 (70.4)
GII.2/GII.2 mixed*	4 (2.3)	1 (1.3)	6 (7.9)	9 (3.4)	75 (23.9)
Non-GII.2	171 (97.7)	79 (98.7)	70 (92.1)	253 (96.6)	239 (76.1)
Total acute gastroenteritis	303	249	264	430	446

*Outbreaks involving either only GII.2 or GII.2 with other GI or GII genotypes.

**Table 2:** Settings of norovirus outbreaks, Taiwan, 2016

Setting	GII.2*	Non-GII.2	Total	p value
School	54 (42.2)	74 (57.8)	128	<0.0001
Kindergarten	28 (66.7)	14 (33.3)	42	<0.0001
Elementary school, grades 1–6	18 (31.6)	39 (68.4)	57	0.0294
Junior high, grades 7–9	3 (20)	12 (80)	15	0.064^†^
Senior high, grades, 10–12, and college/university	5 (35.7)	9 (64.3)	14	0.6033^†^
Restaurant†	13 (11.6)	99 (88.4)	112	<0.0001
Hospital	0	7 (100)	7	0.2035^†^
Long-term care facility	1 (3.7)	26 (96.3)	27	0.0101
Prison/military	0	4 (100)	4	0.5759^†^
Other‡	7 (19.4)	29 (80.6)	36	0.5066
Total	75 (23.9)	239 (76.1)	314	

*Comprises outbreaks with either only GII.2 or GII.2 with other GI or GII genotypes. †Includes hotels. ‡Private residences, street food, dormitories, day care centers.

**Figure 2:**
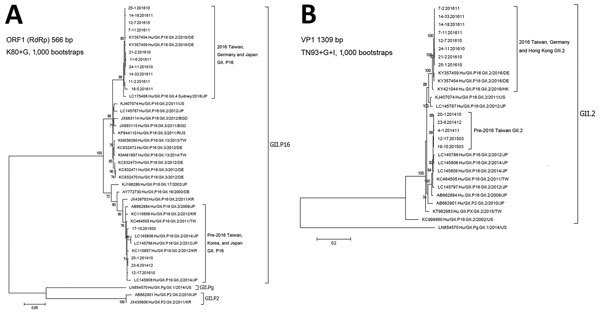
Phylogenetic trees for norovirus GII.2 strains, Taiwan, 2014–2016. A) Partial ORF1 nucleotide sequences in RdRp region (644 nt) of GII.2 strains aligned and the tree generated by using Kimura 80 substitution model with gamma site rates, 1,000 bootstrap replicates, by using MEGA 6.0 software (http://www.megasoftware.net). Bootstrap values of 1,000 replications are shown on the branches. B) Full-length ORF2 nucleotide sequences of GII.2 strains aligned and the tree generated by using Tamura-Nei substitution model with gamma site rates with invariant site using MEGA 6.0 software with 1,000 bootstrap replicates. Bootstrap values of 1,000 replications are shown on the branches. For both trees, norovirus GII.2 reference sequences were downloaded from GenBank; accession number, country, and year are shown. Sequences from Taiwan are indicated by outbreak number and collection year and month. Taiwan GII.2 strains for 2016: 25-1 201610, 14-18 201611, 7-11 201611, 12-7 201610, 24-11 201610, 21-2 201610, 14-33 201611, 11-2 201611, and 18-5 201611; for 2015: 17-10 201503 and 12-17 201503; for 2014: 20-1 20141, 23-8 201412, and 4-1 201411. Corresponding GenBank accession numbers are KY457721–KY457736. BGD, Bangladesh; DE, Germany; HK, Hong Kong; JP, Japan; KR, South Korea; ORF, open reading frame; RdRp, RNA-dependent RNA polymerase; RUS, Russia; TW, Taiwan; US, United States; VP, viral protein. Scale bars indicate nucleotide substitutions per site.

Complete or near full-length open reading frame (ORF) 2 (viral protein [VP] 1) and partial ORF1 (RNA-dependent RNA polymerase [RdRp]) sequences were further obtained from 11 randomly selected Taiwan GII.2 2016 strains by different outbreak and month, as previously described (*2*). However, only 10 and 8 of the RdRp and VP1 sequences, respectively, were of sufficient quality and length for use in analysis. We identified the RdRp region of all GII.2 Taiwan strains as genotype GII.P16 (Figure 2, panel A). Phylogenetic trees were inferred from GII.P16 RdRp and GII.2 VP1 sequences, and in both trees, the GII.2 Taiwan strains form 2 major clusters: 2016 GII.2 Taiwan strains and pre-2016 Taiwan GII.2 strains (Figure 2). Before GII.P16-GII.2 strains appeared, GII.P16 polymerases were found in combination with other strains, such as GII.17 (GenBank accession no. KJ196286), GII.4 Sydney (GenBank accession no. LC175468), GII.10 (GenBank accession no. KC110854), GII.3 (GenBank accession no. KF944110), GII.16 (GenBank accession no. AY682551) in Japan, Korea, and Europe, and GII.13 (GenBank accession no. KM036380) in Taiwan (*9*–*12*). In 2014, GII.P16-GII.2 recombinant strains also were detected sporadically, accounting for only 2 (1.1%) of 170 GII strains in South Korea (*12*). However, these South Korean GII.P16-GII.2 recombinants (GenBank accession nos. KC110856, KC110857) are more similar to the pre-2016 Taiwan GII.P16-GII.2 strains than to the 2016 recombinants in ORF1 phylogenetic tree (Figure 2, panel A). Phylogenetic analysis of VP1 genes of South Korean strains also grouped them with pre-2016 Taiwan GII.P16-GII.2 strains (data not shown). During the 2016–17 norovirus season in Germany, GII.P16-GII.2 strains also were found in nearly half of outbreaks (31/65) and sporadic cases (29/65) (*5*). For the *RdRp* gene, the closest strains to 2016 Taiwan GII.P16-GII.2 strains are the 2016 Germany GII.P16-GII.2 strains (GenBank accession nos. KY357454, KY357459) and a 2016 Japan GII.P16-GII.4 recombinant strain (GenBank accession no. LC175468), which had 98.6%–99.4% identity (Figure 2, panel A) (*10*). In Japan, during the 2004 and 2007–2010 norovirus outbreaks, GII.2 strains mostly were associated with GII.P2 polymerase, and few that had GII.P16 polymerase were classified as recombinants. These recombinants are more closely related to pre-2016 Taiwan strains than to 2016 strains (Figure 2, panel A) (*13*). For the VP1 phylogenetic tree, 2016 Taiwan strains were most closely related to the Germany GII.P16-GII.2 strains (GenBank accession nos. KY357459, KY357454) and a Hong Kong GII.2 strain also sampled in 2016 (GenBank accession no. KY421044), which had 97.8%–99.0% identity, and more distantly to a 2011 US strain (GenBank accession no. KJ407074) and a 2012 Japan (GenBank accession no. LC145787) strain (95% identity for each). The pre-2016 Taiwan strains sampled from 2011–2015 outbreaks that were more closely related to Japan chimeric GII.P16-GII.2 strains that were identified during the 2009–10 and 2012–2014 outbreak seasons in Japan (*13*) (Figure 2, panel B). In both trees, all Taiwan GII.P16-GII.2 strains from 2016 form a cluster that shares close genetic distance (>99% similarity), regardless of the type of transmission or setting of the outbreaks, and the 2016 Taiwan and Germany GII.P16-GII.2 strains are too closely related to discern the difference, suggesting that these 2016 GII.P16-GII.2 strains descended from a recent ancestor that might have been imported and spread within a short time. Furthermore, the close relation of pre-2016 Taiwan GII.P16-GII.2 strains to Japan GII.P16-GII.2 strains and 2016 Taiwan GII.P16-GII.2 strains to 2016 Germany and Hong Kong GII.P16-GII.2 strains could indicate that the pre-2016 and 2016 waves of outbreaks each derived independently from GII.2 strains that were circulating elsewhere globally.

Analysis revealed that RdRp and VP1 of 2016 and pre-2016 Taiwan strains share higher degree of amino acid identity (>98%) than nucleotide identity (91%–93%), indicating more synonymous mutations between the 2 groups. Sites in VP1 that can be used to differentiate between pre-2016 and 2016 Taiwan GII.P16-GII.2 strains were identified as S_71_A, V_335_I, T_344_A, A_354_G, and D_400_E, of which 3 of the sites shared the same residue between the 2016 strains and the 1976 Snow Mountain strain (GenBank accession no. AY134748). However, previous studies have shown that for GII.2 strains, antibodies against the 1976 Snow Mountain strain can block and recognize strains as recent as 2010; thus the GII.2 strains have had only limited evolution in antigenic sites (*14*). Recent 2016 GII.P16-GII.2 strains became the predominant strains of norovirus-associated acute gastroenteritis in Taiwan (Figure 1), although whether they were involved in outbreaks before 2004 is unknown because norovirus monitoring in Taiwan did not begin until that year. The recent outbreaks illustrated the potential of GII.2 to turn from a sporadically detected strain into an unprecedented dominating strain.

## Conclusions

We report the previously uncommon norovirus genotype GII.P16-GII.2 causing an epidemic in Taiwan in late 2016. Previously, GII.2 strains were infrequently reported among both sporadic cases and outbreaks in Taiwan and globally. Before 2014, variants of GII.4, such as New Orleans 2009 and Sydney 2012, followed this paradigm of emergence and rapid replacement every few years, but no non-GII.4 genotypes predominated until the 2014–15 season, when GII.17 emerged in Asia (*4*,*8*). The simultaneous detection and close relation of 2016 Taiwan GII.P16-GII.2 strains with 2016 Germany strains is of importance; given that the previous GII.17 epidemic was limited to Asia, the prevalence of GII.P16-GII.2 should be monitored to assess whether this could become the new predominant strains in other parts of the world. Continued surveillance and unified systems for norovirus typing are critical to monitor the emergence and impact of these and other new norovirus strains.
